# Inhibition of A_2B_ Adenosine Receptor Attenuates Intestinal Injury in a Rat Model of Necrotizing Enterocolitis

**DOI:** 10.1155/2020/1562973

**Published:** 2020-07-03

**Authors:** Lie Huang, Juan Fan, Yan-Xiang Chen, Jian-Hui Wang

**Affiliations:** ^1^Department of Neonatology, The First People's Hospital of Yinchuan, Yinchuan, Ningxia 750001, China; ^2^Department of Neonatology, Ministry of Education Key Laboratory of Child Development and Disorders, National Clinical Research Center for Child Health and Disorders, China International Science and Technology Cooperation Base of Child Development and Critical Disorders, Chongqing Key Laboratory of Pediatrics, Children's Hospital of Chongqing Medical University, Chongqing 400014, China

## Abstract

Necrotizing enterocolitis (NEC) is a lethal gastrointestinal tract disease that occurs in premature infants. Adenosine receptor A_2B_ (A_2B_R) regulates the inflammation cytokine secretion and immune cell infiltration in the colonic pathophysiology conditions. In the present study, we aim to determine the roles of A_2B_R in the development of NEC. A NEC rat model was established and treated with A_2B_R agonist-BAY60-6583 or A_2B_R antagonist-PSB1115. Animals in the control group were free from any interventions. Our results showed that the inhibition of A_2B_R PSB1115 improved intestinal injury and inflammation in newborn NEC rats. The expression levels of caspase-3 and the ratio of apoptotic cells were upregulated in NEC rats, and these indices were downregulated after treating with PSB1115 but further upregulated by BAY60-6583. Meanwhile, a similar trend was also witnessed in the changes of MPO activities and proinflammatory cytokines including IL-6, IFN-*γ*, and TNF-*α*. However, the anti-inflammatory cytokine IL-10 in the NECP group was significantly higher than that in the NEC and NECB groups (*p* < 0.05, respectively). Moreover, the expression of Ki67 was significantly increased in the NECP group as compared with those of the NEC and the NECB groups (*p* < 0.05, respectively). Collectively, our study suggested that the inhibition of A_2B_R attenuates NEC in the neonatal rat, at least partially through the modulation of inflammation and the induction of epithelial cell proliferation.

## 1. Introduction

Necrotizing enterocolitis (NEC) is the most common and lethal gastrointestinal emergency in the neonates. It usually occurs between 27 and 34 weeks after conception, especially in the preterm infants with a very low birth weight < 1000 g [1, 2]. It is still hard to make an accurate diagnosis despite the advanced techniques applied [3]. With the updated modern care and therapy methods, however, overall survival has not changed and the average mortality from NEC is 20-30% [4]. High risk of death or complications make it worth getting more investigations [5, 6].

It was recognized that the increased production of inflammatory mediators, activated receptors which are termed inflammatory cascades, is responsible for the development of NEC[7, 8]. Epithelial injury and intestinal barrier damage were the typical pathological change in NEC, but the underlying mechanism has not been fully understood [9, 10]. The A_2B_ adenosine receptor (A_2B_R) is a transmembrane receptor and is predominantly expressed on the intestinal epithelial cells. Physiologically, it is effective in regulating inflammatory cytokines and limiting immune cell infiltration by triggering adenylyl cyclase activation and phospholipase C activation [11]. Pathologically, A_2B_R was excessively activated such as intestinal ischemia/reperfusion injury [12, 13] or inflammation bowel diseases (IBD) [14–16]. It has been reported that the elevation of A_2B_R as a deleterious consequence may be a target for treatment in IBD [17]. However, a recent study indicated that the specific intestinal epithelial A_2B_R signaling protected the intestines from IBD by enhancing mucosal barrier responses [18]. These nearly contrary conclusions indicate that the function of A_2B_R in the colitis remains controversial.

Different from IBD, bodies suffering from NEC usually owned a more severe mucosal inflammation and intestinal barrier injury. Moreover, the cellular responses to adenosine are varied according to the adenosine receptors expressed, the adenosine concentrations, and the injury type [19]. Until now, there is still lack a research in investigating the role of A_2B_R in the process of NEC. In this study, we aim to investigate the role of A_2B_R in the NEC using its selective agonist and antagonist in rats.

## 2. Materials and Methods

### 2.1. Animals

Specific pathogen-free male SD rats aged 1 day and weighing 5.2–8.4 g were purchased from Cloud-Clone Corp. (Wuhan, China) and housed in cages with a 12 h light-dark cycle for 2 days prior to the start of the experiment. The study was approved by the Animal Experiment Center of the First People's Hospital of Yinchuan (Yinchuan, China). All procedures were carried out in compliance with the Guide for the Care and Use of Laboratory Animals published by the National Institutes of Health (No. 85-23, revised 1996).

### 2.2. Main Reagents and Antibodies

The A_2B_R-selective agonist BAY60-6583 and the A_2B_R-specific antagonist PSB1115 were purchased from Tocris, Bayer Healthcare. The TUNEL detection kit was purchased from Roche Diagnostics GmbH (Penzberg, Germany) and a myeloperoxidase (MPO) colorimetric activity assay kit was purchased from Sigma (MAK 068, USA). The primary antibodies including anti-caspase-3 (ab2302), anti-Ki67 (ab15580), and ELISA measurement kits including IL-6, IL-10, IFN-*γ*, and TNF-*α* were purchased from Abcam (Shanghai, China). The BAY60-6583 was dissolved in 100% dimethyl sulfoxide (DMSO) before being diluted in 0.9% saline. An adenosine assay kit (MET-5090) was purchased from Cell Biolabs, Inc. (San Diego, CA, USA).

### 2.3. Experimental Design and Model Establishment

Rats aged 3 days old were randomly divided into one of the four groups: (1) a control group (control, *n* = 10) without any intervention, (2) a necrotizing enterocolitis group (NEC, *n* = 15), (3) a group of necrotizing enterocolitis with BAY60-6583 treatment (NECB, *n* = 15), and (4) a group of necrotizing enterocolitis with PSB1115 treatment (NECP, *n* = 15). The experimental NEC model establishment has been described previously [20]. Briefly, the model of NEC was established by artificial feeding and hypoxia-cold stimulation. All the animals were housed in an incubator (28-30°C and 45-65% humidity). Rats in the experimental group were artificially fed with the formula substitute of rat milk [21] (4.60 g Liduojing stage I milk powder, 8.68 g albumen powder, 49.2 ml lipid emulsion (C14–24), addition of sterile double-distilled water to 100 ml, and total calories: 581 kJ). The feeding frequency and dosage were set as every 4 h (q4h) at 0.2 ml/time within 24 h, at 0.3 ml/time within 24–48 h, and at 0.4 ml/time within 48–72 h. Additionally, rats in these groups were placed in a vacuum box with pure nitrogen at 25 l/min for 90 seconds and then quickly removed into a refrigerator at 4°C for 10 min for cold stimulation. Subsequently, the suckling rats were removed and returned to the incubator for rewarming, and artificial feeding was continued. Hypoxia-cold stimulation was carried out every 12 h at 10 a.m. and 10 p.m. for 3 consecutive days. Rats in the control group were in the same cage with mothers since birth and fed with rat breast milk. In the drug intervention groups, the A_2B_R-selective agonist BAY60-6583 (1.2-1.25 mg·kg^−1^·d^−1^) [22–24] and the A_2B_R antagonist PSB1115 (1 mg·kg^−1^·d^−1^) [22, 24, 25] were mixed in the formula milk and fed for 3 days continuously. Rats in the NEC group and the control group were administered an equal amount of saline. At 72 h, all the rats were fasted for 12 h and then sacrificed via cervical dislocation.

### 2.4. Sample Collection and Processing

A midline laparotomy was carried out in a sterile environment. The intestinal tube between the lower end of the duodenum and the ileocecal was removed after the mesentery and blood vessel isolation. Then, a 3 cm longitudinal section of the terminal ileum was cut, and the ileum was divided into upper and lower segments. The upper segment was stored in 10% neutral formalin; the lower segment was stored in liquid for measurement.

### 2.5. General Observation

First, animals' daily general state was observed and recorded. Detailed content about these observations has been elaborated by others [26]. Second, the daily disease activity index (DAI) was calculated based on weight loss, stool consistency, and bleeding. The score was calculated as follows. For weight: 0, no loss; 1, 5–10% weight loss; 2, 10–15% weight loss; 3, 15–20% weight loss; and 4, 20% weight loss; for stool: 0, normal; 2, loose stool; and 4, diarrhea; and for bleeding: 0, no blood; 2, presence; and 4, gross blood. Third, variations of the general morphology of intestinal tissues were evaluated after laparotomy. The abdominal cavity was opened, any color or luster changes were noted, and the presence of enteric cavity pneumatosis, necrosis, or hemorrhage was visually checked.

### 2.6. Microscopic Assessment

The intestinal tissues were chopped into 0.5 cm × 0.5 cm × 0.1 cm pieces after 24 h of fixing within a 10% neutral buffer. Samples underwent dehydration and wax immersion and were then embedded in paraffin (ASP200S, Leica, Germany) for cutting into slices (RM2245, Leica, Germany). 5 *μ*m sections were routinely stained with HE (Baso, Wuhan, China). The results were observed using an OLYMPUS IX73 microscope (Olympus Co., Japan). Two double-blind pathology doctors made scorings according to a given standard independently. An assessment of histopathological intestinal damage was performed with the following parameters: morphoarchitectural distortion of intestinal mucosa, villi, and lamina propria and infiltration of the leucocyte. Each of those parameters was scored according to the following scale: 0, the intestinal mucosa and villi were complete and the structure was completely normal; 1, slight submucosal and/or lamina propria separation; 2, moderate submucosal and/or lamina propria separation; submucosal and/or muscular layer edema; 3, severe submucosal and/or lamina propria separation, submucosal and/or muscular layer edema, and local intestinal villi shedding; and 4, intestinal villus disappearance with intestinal necrosis. Leucocyte infiltration was scored as follows: 0, absent; 1, confined to the mucosa; 2, mucosa and submucosa; and 3, traversal of the entire length of the colonic wall. The final scores for each sample were calculated as the sum of those scores.

### 2.7. Immunohistological (IHC) Staining

5 *μ*m sections were prepared for IHC staining. After deparaffinization and rehydration, endogenous peroxidase was inactivated by 3% H_2_O_2_ for 10 min at 37°C. After rinsing in PBS, the sections were incubated for 1 h in blocking solution (5% bovine serum albumin) at room temperature (RT). The sections were then incubated with primary antibody anti-caspase-3 (1 : 500) and anti-Ki67 (1 : 500) overnight at 4°C. After washing three times in PBST (0.1% Tween-20 in PBS), they were incubated with secondary biotinylated goat anti-rabbit antibody (1 : 200) at RT for 2 h followed by enzyme conjugate streptavidin-horseradish peroxidase solution for 1 h. Secondary antibody binding was visualized using 3,3-diaminobenzoic acid dissolved in PBS with the addition of H_2_O_2_ (0.03%) immediately before use. Then, the slides were counterstained with two drops or 100 *μ*l of hematoxylin. Finally, the slides were dehydrated, mounted, and covered with a coverslip. A minimum of three sections per animal were counted for IHC quantitative assessment. Five nonoverlapping fields per section were randomly taken using an OLYMPUS IX73 microscope. ImageJ software was used to count the number of immunopositive cells, which were then averaged per field for each animal. This was blindly performed by an independent, experienced researcher. The values calculated for 10 animals from each experimental group were utilized for comparison and statistical analyses.

### 2.8. Assessment of Cell Apoptosis

The intestinal cell apoptosis was detected using a commercial TUNEL kit according to the manufacturer's instructions (Roche Applied Science). Differential interference contrast microscopy images were obtained at 400x magnification under a fluorescence microscope (OLYMPUS IX73) following the random selection of intestinal mucosa in five nonoverlapping regions. The number of apoptotic intestinal mucosa cells and total intestinal mucosa cells were counted, and then the cell apoptosis rate was determined by the following equation: cell apoptosis rate = the number of apoptotic cells/total cells × 100%.

### 2.9. Measurement of Adenosine Levels in Intestinal Tissues

Intestinal tissue was homogenized in PBS and centrifuged at 10,000 × g for 10 minutes at 4°C. Then, the supernatants were collected and assayed without dilution via the Adenosine Assay Kit MET-5090 (Cell Biolabs, Inc.) according to the manufacturer's instructions.

### 2.10. Myeloperoxidase (MPO) Activity Measurement

MPO activity was measured according to a previously described method [27]. Briefly, intestinal tissue (100 mg) was homogenized in 1 ml of PBS (pH 7.0) containing hexadecyltrimethylammonium bromide (HTAB; 0.5%) and ethylenediaminetetraacetic acid (5 mM, pH 7.4). After centrifugation at 4000 rpm for 15 min at 4°C, the supernatant was assayed in a reaction medium consisting of 50 mM PBS (pH = 7.4) containing 0.167 mg/ml o-dianisidine dihydrochloride and 0.005% H_2_O_2_. MPO activity was measured using a spectrophotometer at 460 nm. One unit of MPO activity was defined as the amount of MPO required to degrade 1 *μ*M hydrogen peroxide (H_2_O_2_) per minute at 25°C. The results of the MPO assay are expressed as units per milligram (U/g) of protein.

### 2.11. Inflammatory Cytokine Measurement

The concentrations of TNF-*α*, IFN-*γ*, IL-6, and IL-10 in the intestinal tissue were measured using enzyme-linked immunosorbent assay kits in accordance with the manufacturer's instructions. Enzyme-linked immunosorbent assays were performed in triplicate.

### 2.12. qPCR

Quantitative polymerase chain reaction (TaqMan probe) and the SYBR-Green I fluorochrome method were used to detect the mRNA expression of TNF-*α*, IFN-*γ*, IL-6, and IL-10. The standard curves of target genes (TNF-*α*, IFN-*γ*, IL-6, and IL-10) and housekeeping genes (GAPDH) were created and used to quantify the expression of the tested mRNAs. Following reverse transcription and amplification, the relative expression amounts of TNF-*α*, IFN-*γ*, IL-6, and IL-10 in each group were determined. The values for each test were normalized using the GAPDH housekeeping mRNA.

### 2.13. Statistical Analysis

Data are shown as a number or mean ± standard deviations (SD). Normal distribution was assessed with the Shapiro-Wilk test. Variables were compared with one-way ANOVA or the Kruskal-Wallis test for nonparametric data. Comparisons between variables within each group were performed with repeated-measurement ANOVA. If there was a significant difference in the overall comparison of groups, comparisons between any other two groups were made by the Bonferroni test. A *p* value of <0.05 was considered statistically significant. The sample rates are compared using Fisher's exact test. The relationship between the parameters was investigated using linear regression and Spearman's correlation. The calculations were performed using GraphPad Prism 8.0.

## 3. Results

### 3.1. Inhibition of A_2B_R Protects NEC Rat against Body Weight Loss

**Figure 1:**
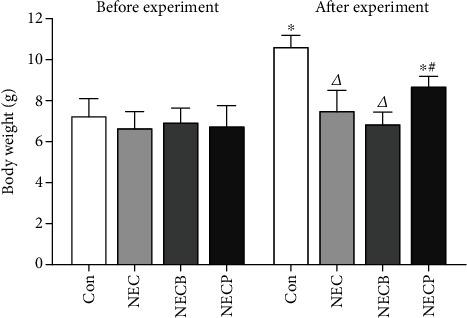
Inhibition of A_2B_R by PSB1115 protected NEC rats against the loss of body weight. No significant differences in body weight were observed between groups before experiment. NEC results in marked body weight loss after experiment as compared to the control group. However, PSB1115 protects NEC animals against body weight loss. NEC: necrotizing enterocolitis; NECB: necrotizing enterocolitis+BAY60-6583 treatment; NECP: necrotizing enterocolitis+PSB1115 treatment. *^Δ^p* < 0.05 vs. all other groups; ^∗^*p* < 0.001 vs. baseline (before experiment); ^#^*p* < 0.001 vs. the Con group.

We randomly assigned 15 rats into each interventional group and 10 rats into the control group. Ultimately, only 40 rats survived the hypoxia-cold stimulation, and the mortality among the interventional groups was insignificant (control: 0/10, NECP: 2/15, NECB: 2/15, and NEC: 1/15, *p* > 0.05). Finally, 13 cases in the NEC group, 13 cases in the NECB group, and 11 cases in the NECP group developed NEC by visual inspection. As seen in Figure 1, the average body weight after the experiment was significantly decreased in the NEC, NECB, and NECP groups as compared with the control group (*p* < 0.001, respectively). However, the body weight in the NEC and NECB groups was significantly lower than that in the NECP group (*p* < 0.001, respectively). In addition, animals in the NECB group were further decreased as compared with those in the NEC group (*p* < 0.05).

### 3.2. Inhibition of A_2B_R Attenuates NEC in Newborn Rat

**Figure 2:**
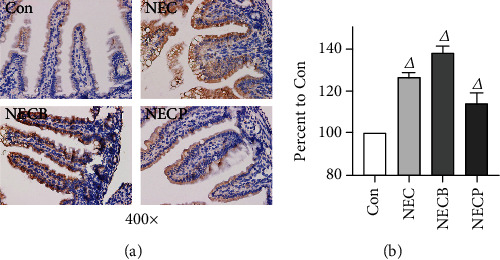
A_2B_R expression levels during NEC between groups. (a) A_2B_R expression in intestinal tissues was determined by IHC staining (400x). (b) Quantitative changes of A_2B_R expression. NEC: necrotizing enterocolitis; NECB: necrotizing enterocolitis+BAY60-6583 treatment; NECP: necrotizing enterocolitis+PSB1115 treatment. *^Δ^p* < 0.05 vs. all other groups.

**Figure 3:**
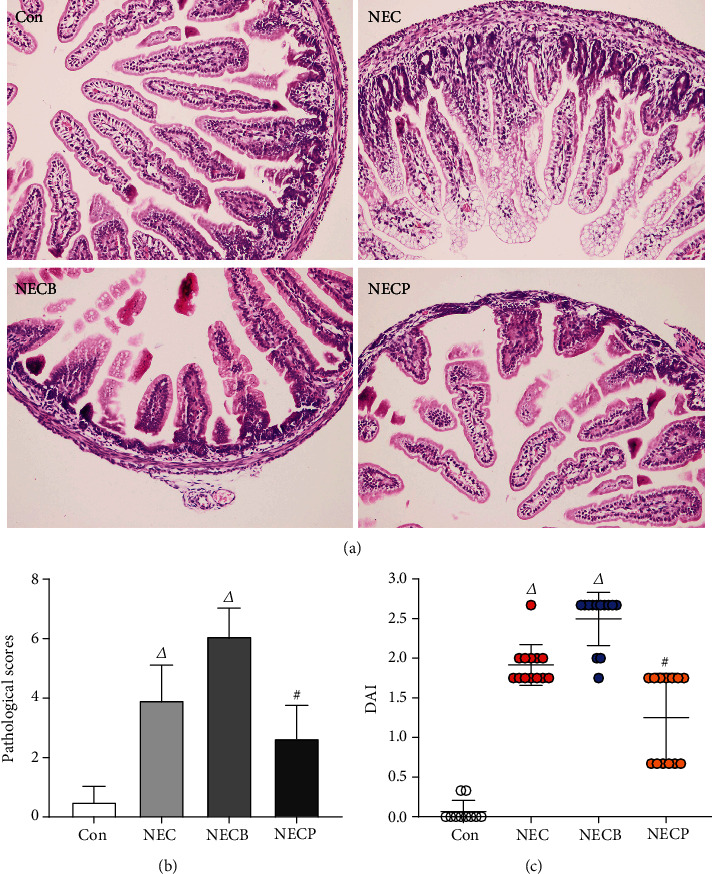
Inhibition of A_2B_R by PSB1115 attenuated NEC. (a) H&E staining (200x) of intestinal tissues showed improved morphology in the NECP group as compared with the NEC and NECB groups; (b) pathological scores in the NECP group were significantly lower than that in the NEC and NECB groups; (c) the disease severity of NEC was determined by the DAI score. Animals in the NECP group showed lower DAI scores than those in the NEC and NECB groups. DAI: disease activity index; NEC: necrotizing enterocolitis; NECB: necrotizing enterocolitis+BAY60-6583 treatment; NECP: necrotizing enterocolitis+PSB1115 treatment. *^Δ^p* < 0.05 vs. all other groups; ^#^*p* < 0.01 vs. the Con group.

Firstly, we measured the expression levels of A_2B_R during NEC by IHC, and we found that A_2B_R expression was upregulated in the NEC group, which was more significant in the NECB group when compared to the control. However, the upregulation of A_2B_R was significantly inhibited in the NECP group (Figure 2). These results indicated that the intestinal A_2B_R was activated when NEC occurred and could be effectively inhibited by PSB1115 administration. The microscopic observations of intestinal tissues are shown in Figure 3(a). Compared to the control group, animals in the NEC group exhibited surface mucosal ulceration with necrosis. Specifically, a moderate or even severe submucosal and lamina propria separation accompanied with moderate inflammatory cell infiltration and villi edema widely existed in the tissue. The leucocyte infiltration was most limited in the mucosa and submucosa. In the NECB group, intestinal morphology with necrosis changes was more prominent than in the NEC group. Besides a severe mucosal separation, villi edema, and leucocyte infiltration, construction of the mucosal, submucosal, and villi even disappeared in some parts. Interestingly, significant necrosis morphological changes which occurred in the NEC and NECB groups were not observed in the NECP group.

The hypoxia-cold stimulation resulted in a significant increase in the histological scores in all the interventional groups compared to the control (*p* < 0.001) (Figure 3(b)). The histological scores in the NECP (2.64 ± 1.12) group was significantly lower than those in the NEC group (3.92 ± 1.19, *p* < 0.001) and the NECB group (6.08 ± 0.95, *p* < 0.001).

The DAI score was used to assess the disease severity. At the end of the experiments, DAI scores were significantly higher in the NEC, NECB, and NECP groups as compared with the control group (*p* < 0.05, respectively) (Figure 3(c)). The DAI scores in the NECP group were significantly lower than those in the NEC and NECB groups (1.25 ± 0.56 vs. 1.92 ± 0.26 and 2.50 ± 0.34, *p* < 0.01, respectively). In addition, the DAI scores in the NECB group were significantly higher than those in the NEC group (2.50 ± 0.34 vs. 1.92 ± 0.26, *p* < 0.05, respectively).

### 3.3. Inhibition of A_2B_R Decreases MPO Activity

**Figure 4:**
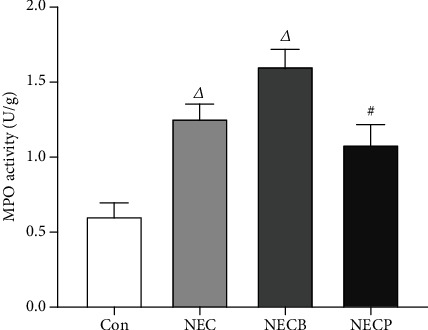
Inhibition of A_2B_R by PSB1115 downregulated MPO activity in NEC rats. MPO activities were determined in all animals at the end of experiments. Animals in the NECP group showed significantly lower MPO activity as compared with those in the NEC and NECB groups. NEC: necrotizing enterocolitis; NECB: necrotizing enterocolitis+BAY60-6583 treatment; NECP: necrotizing enterocolitis+PSB1115 treatment. *^Δ^p* < 0.001 vs. all other groups; ^#^*p* < 0.001 vs. the Con group.

As seen in Figure 4, a significantly increased MPO activity was observed in the NEC and NECB groups as compared with the control group (*p* < 0.01). The NECB group showed a markedly increased MPO activity as compared with the NEC group (1.60 ± 0.11 vs. 1.26 ± 0.10 U/g, *p* < 0.01, respectively). However, MPO activity in the NECP group was significantly lower than that in the NEC group (1.08 ± 0.13 vs. 1.26 ± 0.10 U/g, *p* < 0.01, respectively).

### 3.4. Inhibition of A_2B_R Downregulates Inflammatory Cytokine Production

**Figure 5:**
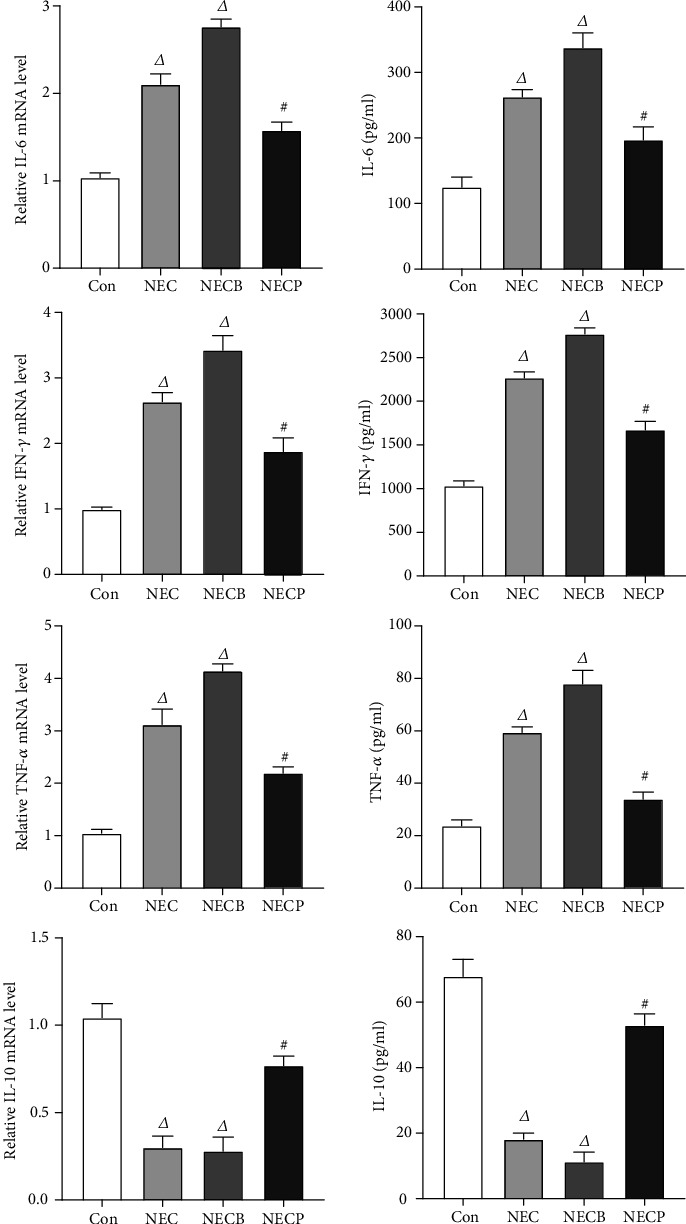
Inhibition of A_2B_R by PSB1115 attenuated inflammation in NEC rats. Proinflammatory (IL-6, IFN-*γ*, and TNF-*α*) and anti-inflammatory (IL-10) cytokines were measured by ELISA assays. NEC: necrotizing enterocolitis; NECB: necrotizing enterocolitis+BAY60-6583 treatment; NECP: necrotizing enterocolitis+PSB1115 treatment. *^Δ^p* < 0.01 vs. all other groups; ^#^*p* < 0.001 vs. the Con group.

The antagonist PSB1115 could effectively downregulate proinflammatory cytokines that statically increased in the NEC and NECB groups. The intestinal levels of TNF-*α*, IFN-*γ*, and IL-6 were relatively lower in the NECP group compared to the NEC group (Figure 5) (*p* < 0.05). Contrarily, the level of IL-10 which decreased in the NEC group and the NECB group was significantly increased in the NECP group (*p* < 0.05). Meanwhile, a similar trend was witnessed in the changes of mRNA expression levels of these cytokines.

### 3.5. Inhibition of A_2B_R Decreases Apoptosis and Promotes Epithelial Cell Proliferation in Neonatal NEC Rats

**Figure 6:**
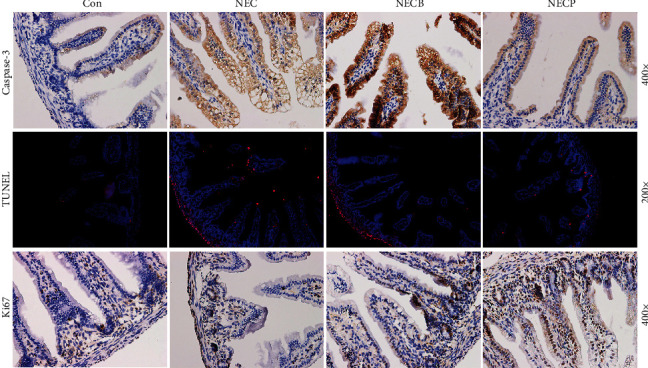
Inhibition of A_2B_R by PSB1115 decreased apoptosis but promoted epithelial cell proliferation. Cell apoptosis in intestinal tissues was determined by IHC staining for caspase-3 (a) and TUNEL assay (b). In addition, Ki67 staining was performed to assess cell proliferation. NEC: necrotizing enterocolitis; NECB: necrotizing enterocolitis+BAY60-6583 treatment; NECP: necrotizing enterocolitis+PSB1115 treatment.

As seen in Figure 6, cell apoptosis was determined by IHC staining of caspase-3 and TUNEL assays. The number of caspase-3-positive cells in the NECP group was increased compared with those in the NEC and NECB groups. In addition, TUNEL assays showed that the ratio of apoptotic cells in the NECP group was significantly higher than that in the NEC and NECB groups (12.6 ± 5.5% vs. 35.6 ± 12.7% and 42.1 ± 9.4%, *p* < 0.05, respectively). Moreover, the cell apoptosis rate in the NECB group was upregulated compared with that in the NEC group (42.1 ± 9.4% vs. 35.6 ± 12.7%, *p* < 0.05, respectively). In the present study, Ki67 was stained to assess cell proliferation in intestinal tissue. Our results showed that Ki67-positive cells in the NECP group were increased compared with those in the NEC and NECB groups (39.7 ± 14.2 vs. 22.3 ± 8.2 and 19.2 ± 7.5 cells/per field, *p* < 0.05, respectively).

## 4. Discussion

The present study was designed to determine the role of A_2B_R in the development of NEC, which resulted in a remarkable activation of intestinal A_2B_R in intestinal tissues. The inhibition of A_2B_R activation improves NEC together with decreased inflammation cytokines and neutrophil infiltration. In addition, cell apoptosis and epithelial proliferation were also downregulated by the administration of the A_2B_R antagonist. Our results showed that the inhibition of A_2B_R was efficient in preventing intestinal injury against NEC, which was possible through the downregulation of inflammation.

The first and major finding of our study was that the A_2B_R agonist failed to improve but aggravated the intestinal histological injury that was already impaired during NEC after hypoxia-cold stimulation. A complete intestinal structure is important for water and nutrient absorption, microbial colonization maintenance, and waste product transportation in the rhythmically activated intestine tract. However, NEC resulted in partial or total mucosal injury, especially at the inner and middle mucosa [28]. We found colonic tissue ulceration, submucosal edema, loss of epithelial lining and goblet cells, and inflammatory cell infiltration in the mucosa and submucosa. Administration of BAY60-6583 resulted in aggravation of the ulcerations with more epithelial loss, severe goblet cell loss, deterioration in submucosal edema, and severe inflammatory infiltration of the submucosa. This part of our finding is supported by other models. It was reported that activation of A_2B_R on intestinal epithelial cells could augment IL-6 production and increased neutrophil activation combined with increased detection of A_2B_R in epithelial cells isolated from the mouse or human colitis [29, 30].

The second major finding was that the A_2B_R antagonist PSB1115 alleviated the intestinal structural injury and neutrophil infiltration and decreased cell apoptosis. In our study, a worsening of intestinal injury was witnessed in the NECP group that was treated with BAY60-6583. However, these changes were attenuated in the NECB group that was administrated with PSB1115. Accompanied with pathological changes, some major inflammatory cytokines such as IL-6, TNF-*α*, and IFN-*γ* were also increased under NEC condition. Different from A_2A_R, A_2B_R is predominantly expressed on the intestinal epithelial cells [31]. It is effective in regulating inflammatory cytokines and limiting immune cell infiltration in intestinal diseases. Lots of researches revealed that the A_2B_R signaling played a beneficial role in protecting intestinal mucosa during inflammation via enhancing mucosal barrier responses [32–34]. For example, A_2B_R^−/−^ mice experienced a significantly heightened severity of colitis, associated with a more acute onset of disease and loss of intestinal epithelial barrier function [18]. However, it was also reported that adenosine signaling via the A_2B_R aggravated mucosal inflammation and tissue injury during experimental colitis [17, 35]. In fact, the cellular responses to adenosine are varied according to the adenosine receptors expressed, the adenosine concentrations, and the injury type [19]. But the reason which caused these nearly contrary conclusions remains unclear. We established a neonatal animal model to test the effect of A_2B_R on NEC. Our result indicated that the activation of A_2B_R induced an increased production of proinflammatory cytokines and aggravated mucosa injury under NEC, at least in preterm.

Another key finding of our study was that the A_2B_R antagonist PSB1115 intervention promoted the proliferation of epithelial cells under the NEC condition. It is generally recognized that loss of normal intestinal barrier function is fundamental to the pathophysiology of NEC [36]. The integrity of the intestinal barrier is maintained mainly by tight junctions which are composed mostly of endothelial and epithelial cells. The destruction in either the endothelial or the epithelial intestinal barrier could accelerate the development of NEC. Increased permeability of the epithelial barrier allowed for increased flux of inflammatory mediators, bacterial translocation, and breakdown of the intestinal architecture which would further aggravate the mucosal barrier dysfunction [37]. All these changes induced epithelial cell edema, apoptosis, and even death. It has been reported that the enhanced epithelial cell proliferation was associated with an alleviated NEC severity [38]. The proliferated epithelial cells contributed mostly in the repair of the impaired mucosa barrier, and we observed increased epithelial cell proliferation in NEC rats after PSB1115 administration.

There were some limitations to this study. First, the expression of caspase-3 and Ki67 in the intestinal tissue was not quantified by western blot. These results were semiquantified in our research. Second, the physiological data such as body temperature, heart rate, and blood pressure were not measured because the animals are too small. Third, rats in the control group were fed with mother's milk. The potential mixture factor caused by milk feeding could not be avoided. Finally, this study was conducted on healthy rats free of underlying diseases, and the direct applicability to humans is not assumed.

## 5. Conclusions

The enteral administration of A_2B_R adenosine receptor antagonist-PSB1115 rather than agonist-BAY60-6583 can attenuate the intestinal injury in the neonatal NEC rat, possibly through the regulation of the inflammatory process and the induction of epithelial cell proliferation.

## Data Availability

The data were available from the corresponding author on reasonable request.
